# The effect of educational computer games on the academic resilience, academic self-regulation, and academic achievement of EFL students

**DOI:** 10.3389/fpsyg.2022.947577

**Published:** 2023-01-23

**Authors:** Lin Deng, Nikoo Daverpanah, Siros Izadpanah

**Affiliations:** ^1^College of Xingzhi, Zhejiang Normal University, Zhejiang, China; ^2^Department of English Language Teaching, Zanjan Branch, Islamic Azad University, Zanjan, Iran

**Keywords:** computer games, academic resilience, academic self-regulation, academic achievement, learning

## Abstract

**Introduction:**

In recent years there has been an increasing interest in the field of educational computer games (ECGs). Although ECGs have been researched, more analyses still need to be performed on these variables to check their effects on language learning.

**Methods:**

To this end, 74-third grade female state high school students from two schools in Zanjan were selected through a two-stage cluster random sampling method. The number of students in each class was 37. One of the classes (control group) was trained traditionally and the other was for one semester through the researchers-made ECGs. After completing the training, the research tools were performed as a post-test on the experimental and control groups. The data collection stage took place for about 6 months.

**Results:**

Based on the results from the research questions, the use of training computers has been effective in increasing AR, ASR, and AA.

**Discussion:**

It has significant implications for teachers and learners in the EFL context and opens interesting opportunities for administrators and curriculum developers to explore when planning EFL courses.

## Introduction

One of the apparent effects of the world is the use of new technologies. In recent decades, traditional learning approaches have undergone fundamental changes (Sife et al., 2007; Jantakun and Jantakoon, 2021). Investigation of the factors that influenced the progress, development, and advancement in developing countries indicates that all these countries have capable and efficient education. Scientific and technological advances in today’s world and the speed of changes in the fashions and scientific methods are very rapid and astonishing (Lepper and Malone, 2021). The growing need for people to education and related problems to access education in the traditional way has been developed by experts to invent new educational technologies (Haque and Sharif, 2021; Hooshyar et al., 2021). In today’s world, increasing digital media and technologies have influenced teachers’ and students’ activities (Zimmer et al., 2021). One of the issues that have attracted a lot of attention today is computer skills at different levels of education. Today’s learners are from a different generation, a different generation is surrounded by computers, games, video cameras, mobile phones, and other digital tools. This generation can be called “a generation of network,” “digital generation,” or “digital indigenous” (Taheri et al., 2021).

Compared to this group, people who have not been born in the digital age and only adapted to technology are called “digital immigrants.” The most challenging problem with today’s education is the obsolete immigrant teachers who are related to the early digital era; they are trying to teach people who speak a new language (Deta et al., 2021). Therefore, it should be noted that using a common language is to educate these individuals, and new technologies can bring this common language. Digital games can be applied to the learning method of today’s students. It has been critical in recent years, and its educational skills have become noteworthy (Shakarboevna, 2021).

The purpose of any educational system is to increase students’ ability in general. The realization index or non-realization is learners’ academic achievement (Lim et al., 2022). Various factors can be influential on academic achievement. According to (Kermarrec et al., 2021), students entertained at home with computer programs have better academic achievement. Further research has shown the effectiveness of computer games on different variables (Lepper and Malone, 2021; Abd-Alrazaq et al., 2022). Various researches have shown the effectiveness of computer games in different variables. Considering the problems related to teaching English in Iranian schools and the fact that today’s students are from a generation that is taught and speaks another language, it seems that technology and in particular educational computer games to be effective on academic resilience, academic self-regulation, and academic achievement on EFL students. Thus, this study aimed to investigate the effect of educational computer games on academic resilience, academic self-regulation, and academic achievement of EFL Iranian female students in the third grade of high school.

## Review of the literature

### Educational computer games

It is stated that educational computer games consider a specific subject or educational content of a particular course and can be suitable for any type of learning environment (Mubireek, 2003). Kaimara et al. (2022) also point out that educational computer games can be used for all four types of educational purposes such as 1. Practicing and rebuilding knowledge of skills already acquired. 2- Identifying gaps and weaknesses in the knowledge and skills of the individual. 3- Reviewing or summarizing the existing knowledge and skills of the person before holding an exam. 4. Creating relationships between concepts and principles. These goals are not necessarily independent of each other. In addition to reviewing and summarizing, a game may create new relationships between concepts and principles.

Undoubtedly, the world is progressing quickly, and this growth is more dramatic in terms of technology. Today, many aspects of computer have become one of the most critical aspects of the game. Computer game technology has attracted a wide range of people and has become very popular (Erbas et al., 2021; Ghory and Ghafory, 2021). These games refer to games played through personal computers and performing them requires fast information processing and providing logical and extremely fast information. In other words, a computer game is a program that is a set of instructions and allows players to interact. It shows that they can come together and lead to a specific result. Alt and Naamati-Schneider (2021) believe that computers are a form of entertainment designed to achieve specific goals and have specific rules. However, it is inconsistent with Kim and Chang (2010) research. This inconsistency can be a computer game and was not an educational one.

Computer games are about 60 years old and have been around since the 1960s. In 1962, Elstow Rusell, a student at MIT, designed the first computer game called Space War. It was not until the 1970s that computer games were introduced into handheld technologies (Geisel et al., 2021). Most computer manufacturers were in the United States and Japan. It was not long before computers became a popular pastime among children, teens, and adults alike. In the early 1990s, the Internet provided a new way of offering games (Jantakun and Jantakoon, 2021). Children and adults were able to play their favorite games online. These games are a more advanced type of computer game because these games are played in groups. Gamers establish more social connections with other people.

### Academic self-regulation

Academic self-regulation is one of the concepts in the education of the contemporary world, which today is considered an important center and one of the primary axes of education (Hirt et al., 2021; Luo, 2021). Academic self-regulation has been increasingly used in learning various skills, including cognitive-motor and social skills.

Staller et al. (2021) refer to self-regulated learning strategies as a type of learning in which learners initiate and direct their efforts instead of relying on parents or other educators for knowledge and skills. In other words, in their view, self-regulation in learning can be considered the learner’s active participation in cognitive and metacognitive motivational behavior. Such an ability allows a person to be in control of their behaviors. That is, evaluate his behaviors, measure them by their standards, and apply reinforcement and punishment to themselves. Elsewhere, self-regulatory learning is a type of learning in which one tries to begin and direct one’s efforts to acquire knowledge and skills without relying on the teacher and the other person. In other words, people develop skills to design and control their learning process and are willing to learn while evaluating it.

The self-regulatory structure of education refers to strategies that enable individuals to demonstrate the direction of their goals in the development process (Zimmerman and Schunk, 2001). In other words, self-regulatory learning and empowering students to provide opportunities to actively manage processes such as setting self-assessment goals, self-assessment, and motivation. Raković et al. (2022) believe that self-regulation is the ability to think and solve problems without the help of others.

Bransen et al. (2022) describe self-regulation as the perception of individuals of their ability to design and perform essential behaviors to achieve specific goals. Most definitions of self-regulation is that most historians assume that learners are aware of the potential benefits of self-regulatory learning processes in enhancing their academic achievement. Feedback loops during learning are other features of the definitions. This loop is a cyclical process in which learners monitor the effectiveness of their learning strategies and methods. The third feature describes the situation and how learners choose specific strategies or self-regulatory processes.

### Academic resilience

Today, the educational system’s concern is creating a suitable environment for the growth and excellence of intellectual capital in a knowledge-based society (Meneghel et al., 2019). For all social groups to be able to participate effectively in such a community, they must learn creativity, innovation, as well as active and constructive social participation. Therefore, the use of educational games in education is the basis for motivation, learning, experience, and its application in education. Education is expected to provide active and participatory learning among students. It is necessary to change the previous procedures to achieve such an approach. Old-fashioned teaching methods certainly do not meet the changing educational needs of the modern age; thus, the efforts of educational organizations should be related to information and communication technology and its application in the curriculum (Singh, 2021; Hunsu et al., 2022).

Technology brings the benefits of computer games to school for administrators, executives, and educators and enables students to learn computer game literacy in the early stages of their lives. Instead of learning individually, students use new technologies to establish communication and interact with each other in school. Talented students learn faster, they can move on to newer and more advanced subjects, and weaker ones continue to learn until they are ready to move on to the next step (Ononye et al., 2022). Academic resilience is one of the most important topics that has attracted the attention of many sociologists, psychologists, and educators; because academic resilience is one of the important factors that strongly affect academic achievement and performance. Academic resilience is a particular branch of the general concept of adaptation that deals with the issue of individual adaptation to the course, field of study, educational environment, and requirements.

### Academic achievement

Assessment of academic achievement in general and academic achievement in English as a foreign language or second language (achievement English ESL / EFL) in particular, as well as their determinants, have attracted the attention of experts and researchers in the field of education for many years (Balim et al., 2020; Şentürk, 2021). Much of the theoretical discussion and practical research conducted in second language learning has been aimed at finding answers to why some learners succeed in learning foreign languages but others do not perform well? In today’s world, academic achievement is of particular importance. Advanced societies emphasize the performance of competition and victory (Abuhassna et al., 2020; Ayra and Kösterelioglu, 2021). One of the most critical and objective criteria for evaluating the effectiveness of education systems is the academic achievement of students (Zeynali et al., 2019; Gökalp, 2020). Wang et al. (2018) refer to all student conflicts in the school environment, including the self-efficacy of emotional effects. Planning is a lack of control over outcome and motivation. In other words, the term refers to students’ success in one or more subjects such as understanding comprehension, reading numerical calculations, etc. Academic tests measure these improvements. The concept relates to the progress of individuals in the classroom as assessed in schoolwork can be applied (Guo et al., 2021).

### Iranian EFL computer games

Teaching a foreign language is a topic that is becoming more important day by day. In many countries, this has allocated an essential part of educational facilities. English language course has encountered lots of challenges in Iran. Despite spending a lot of time and money on English courses, it is very observable that students cannot read and write simple text in English. One of the reasons for this failure is the common teaching methods in the country’s educational system (Shute et al., 2021). Teachers are always looking for the best educational methods to help students be interested and successful in understanding the subjects presented in the classroom (Saif, 2005).

On the other hand, teachers are always concerned about increasing students’ academic resilience and learning at all levels of education. One of the dimensions of self-regulation is self-regulation in learning and study. Organizing and regulating the main learning processes and their activities are carried out through academic self-regulation. Self-regulation has a positive relationship with academic achievement (Alt and Naamati-Schneider, 2021). Students learn skills to design, control, and evaluate the learning and educational process using educational skills (Zimberman and Martchins, 2002).

Considering the problems related to teaching English in Iranian schools and the fact that today’s students are from a generation that is taught and spoke another language, it seems that the use of technology, and in particular educational computers to be more effective. Thus, this research aims to study educational computer games on academic self-regulation - academic resilience and academic achievement in Zanjan, Iran.

This research studies these hypotheses:

1. Computer educational games significantly affect the academic resilience of third grade (twelfth) female students in Zanjan, Iran.
2. Computer educational games significantly affect the academic self-regulation of third grade (twelfth) female students in Zanjan, Iran.
3. Computer educational games significantly affect the academic achievement of third grade (twelfth) female students in Zanjan, Iran.

### Iranian and international studies on educational computer games on academic resilience, academic self-regulation, and academic achievement on EFL students

Katsarou (2021), in the study of the relationship between computer anxiety and computer self-efficacy, the findings showed that computer self-efficacy, computer experience and motivation for progress have the most negative and significant relationships with computer anxiety.

Rahimy and Hoseinzade (2021) examined the relationship between Gardner’s multiple intelligences and anxiety, computer self-efficacy, and the results showed that there is a positive and significant relationship between the components of multiple intelligences and computer self-efficacy.

Biduri et al. (2021, July) examined the relationship between computer self-efficacy, computer anxiety, and attitudes toward the Internet, the results showed that people with moderate anxiety and moderate attitudes toward the Internet had higher levels of computer self-efficacy.

Taheri et al. (2021), in the study of the relationship between creativity and academic achievement, showed that there is a positive and significant relationship between the components of creativity and academic achievement.

Liu et al. (2022) examined the function of computer games in adolescent behavior, the results showed that there is no difference between the mental ability of students who use computer games compared to students who do not use it.

Cheah et al. (2022) examined the effect of computer games on the development and motivation of learning geography in primary school children and showed that computer games can be used as formal educational tools for this course in formal educational environments. The Center for Media Studies, after 2 years, concluded that computer games improve adolescents’ reading and enable them to solve problems.

## Materials and methods

### Design of study

The present study was quasi-experimental with pre-test and post-test design and a control group.

### Statistical population

In this study, the statistical population includes 665 female students who studying in the third grade of high school in Zanjan, Iran. Inclusion criteria were female gender, studying in third grade of high school, a high school without tuition fees, and willingness to participate in the study. There are 38 high schools in Zanjan city; out of them, 19 high schools are for females. Out of 19 high schools, 10 high schools met inclusion criteria. In this study, eligible participants were selected using the multi-stage sampling method in this way. Level 1: The eligible high schools were considered as a cluster. Level 2: Two high schools were selected randomly and assigned to experimental and control groups. Level 3: Each selected high school (experimental /control groups) had two classes in third grade. Out of these, one class was randomly selected in each group. The number of students in each class was 37 students, as a result total sample size was 74 students.

### Research instruments

**Figure 1:**
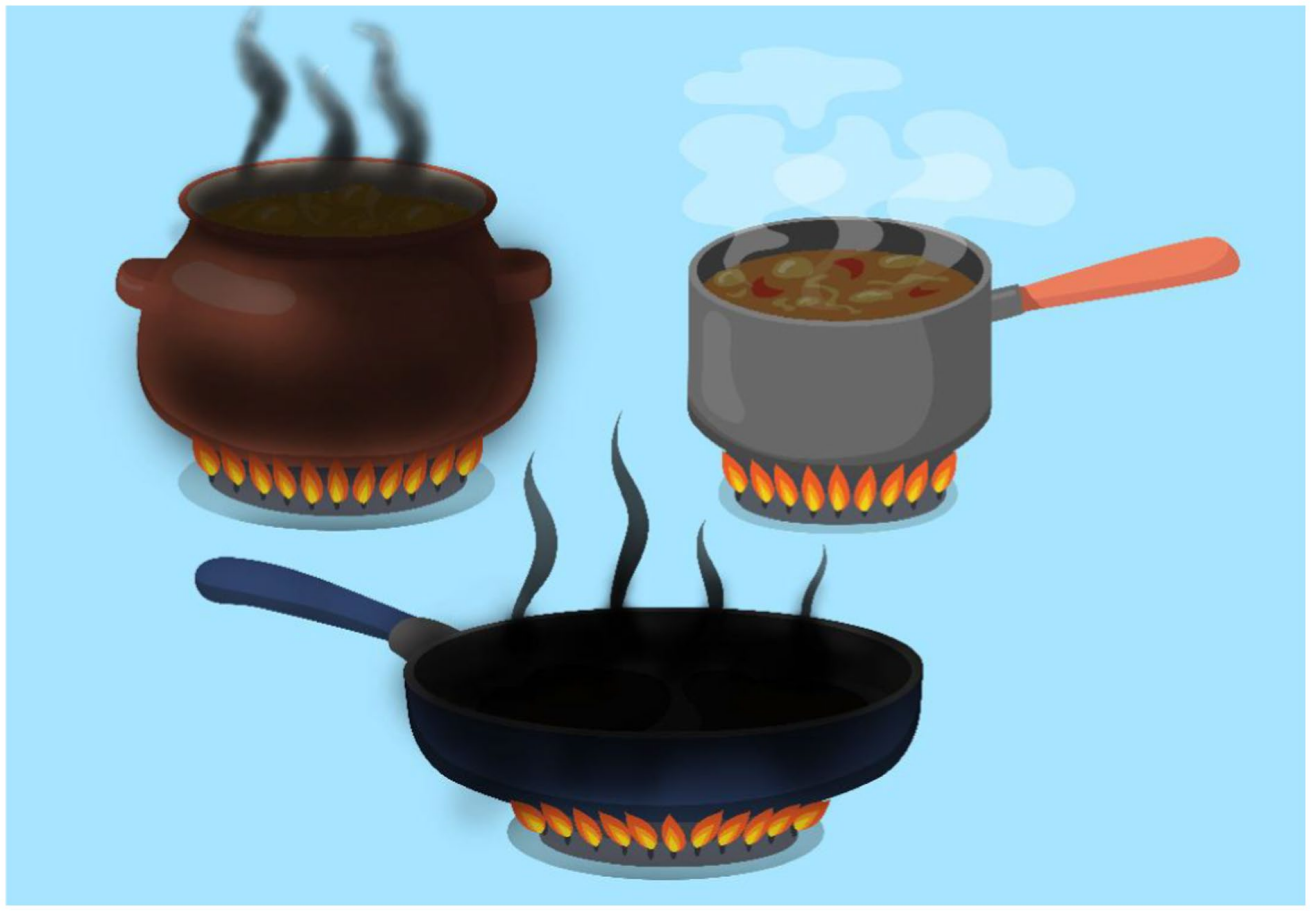
Results of the correct and wrong answers.

**Figure 2:**
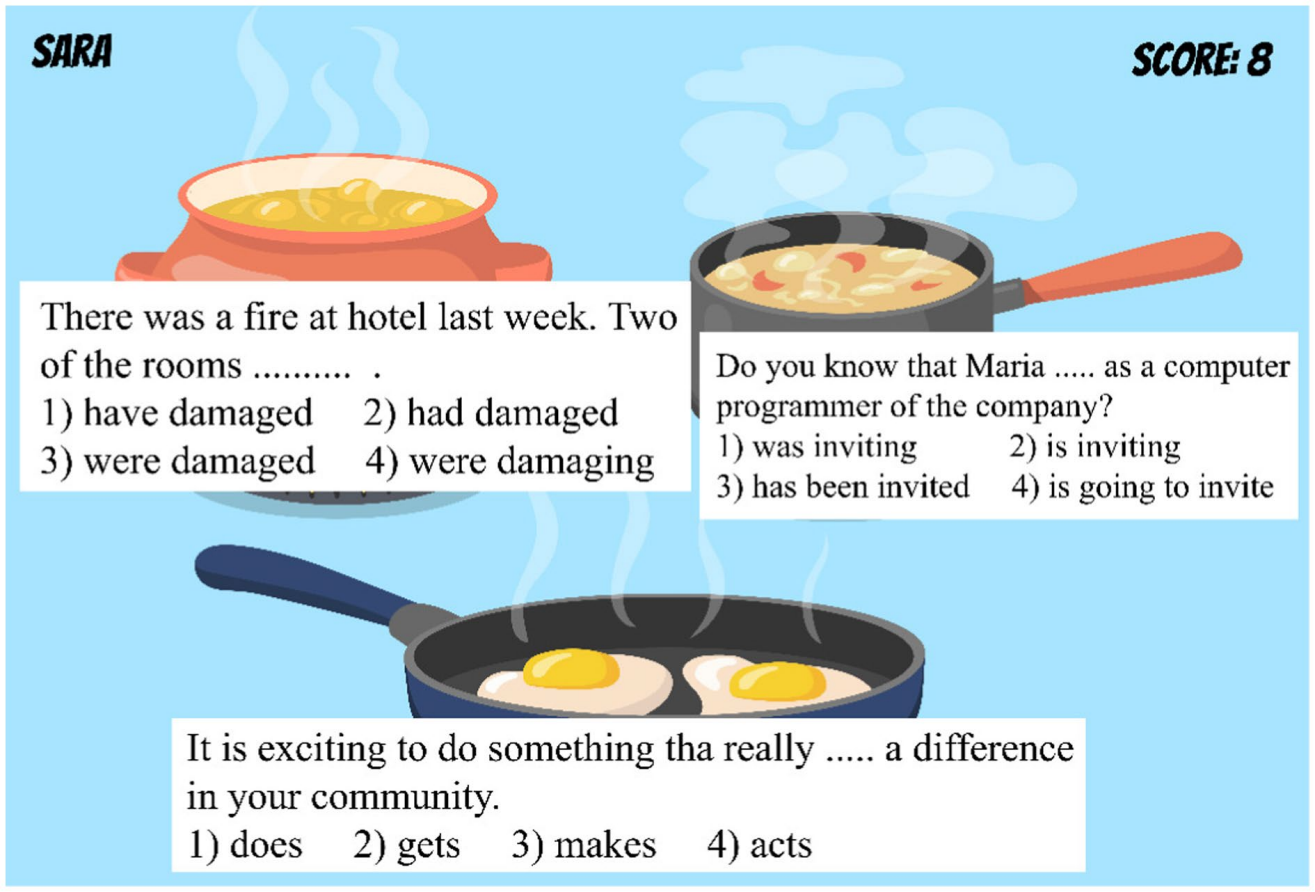
Questioning the students.

In this study, the researchers have made a computer game using Adobe Flash and ActionScript. ActionScript is the programming language for the Adobe^®^ Flash^®^ Player and Adobe^®^ AIR^™^ run-time environments. It enables interactivity, data handling, and much more in Flash, Flex, and AIR content and applications. ActionScript executes in the ActionScript Virtual Machine (AVM), which is part of Flash Player and AIR. This game has been designed in two sections: education and preparation for English lessons for third-grade high school students. First, the students should pass the first section, including multimedia training, and then they can enter the game. In the game, the students were asked questions and were given appropriate feedback. If the learner provides the correct answer to the game questions, the food does not burn, and if the answer is wrong, the food will burn. Finally, the student receives general feedback on the success or failure to play (Figures 1,2).

### Results of the right and wrong answers

The student answered two questions incorrectly and 1 question correctly. Two burned foods are the result of the wrong answers and one well-cooked food is the result of the correct answer. If all the answers were correct, all three pictures would appear as cooked food.

### Questioning the students

Some questions are presented to the students to score them. At every level, the difficulty of the questions will increase. Before answering the questions, the food appears raw and if the answer is right, then the food will be well cooked in the next phase. If the answer is not right, the food will appear burned. This picture shows the fourth level; the student (in this case Sara) answered 8 of 9 questions correctly.

### Using adobe flash and ActionScript to create the game

Photoshop was used to create the pictures used in the game. Each picture for each food was designed three times (raw, cooked, burned). Adobe Flash and ActionScript are used to create the game using the photos designed in photoshop and different questions with different difficulty levels.

### Questionnaire reliability

Regarding the reliability of the questionnaires, Cronbach’s alpha index obtained for the questionnaires of academic resilience is equal to 0.904, academic self-regulation is equal to 0.716, academic achievement is equivalent to 0.734, and greater than 0.7, the questionnaires have the necessary reliability.

### Instruments

#### Academic resilience

To collect data from the standard questionnaire of academic resilience of Samuels (2004), which was standardized by Soltaninejad et al. (2013) was used. It has three components: “communication skills,” “future and problem-oriented orientation,” and “positivity” and 55 items with a five-point Likert scale (from never with a score of 1 to always with a score of 5). Its face and content validity by 10 professors in the English language department was approved. The reliability of the questionnaire was calculated through Cronbach’s alpha coefficient of 0.81.

### Bofard’s self-regulation

The 14-item questionnaire by Bofard et al. (1995) is a self-regulatory assessment tool based on Bandura’s social cognitive theory. Questions are on the Likert scale and measure two factors of cognitive and metacognitive strategies (quoted by Kadivar, 2001). The scoring method using the Likert scale is from strongly agree (score 5) to disagree (score 1) strongly, and questions 5–13-14 are scored in reverse. Kadivar (2001) has studied the validity and reliability of this instrument. The construct validity of this questionnaire was reported to be optimal by using correlation coefficients and factor analysis of correlation coefficients between the questions of the questionnaire, and Cronbach’s alpha coefficient for measuring internal consistency was 0.08. Based on this, it can be said that this questionnaire can predict the actual scores of the subjects.

### Academic achievement

This questionnaire consists of 48 questions from Pham and Taylor’s (1994) standard questionnaire, classified as a 5-point Likert scale from very high with code 5 to none with code 0. The highest score is 240, and the lowest score is 48. Moradian (2013) has confirmed its validity through content validity by experts and specialists. Moradian (2013) obtained the reliability of the questionnaire using Cronbach’s alpha coefficient of 0.82.

### Language learning games

Language learning games for the classroom are one of the best ways to promote language learning. There is an old Chinese proverb that says: “tell me, and I will forget. Show me, and I may remember. Involve me, and I will understand” and games are one of the ways to involve students in a fun learning environment.

This game is reward-based in the sense that it uses positive reinforcement to achieve what we call the right answer. It is a valid protocol to motivate students to eliminate wrong answers. This is a simple example of how positive and negative reinforcement can work together. Of course, this does not mean that every aspect of language can be taught this way but it is more of a motivator. More importantly, it sands off the rough edges of fossilized wrong language knowledge and cultural barriers. Players (students) are presented with several questions and for each question, there is a specific picture, not necessarily related to the topic. Based on the answer, if correct then the food will be presented as well cooked, and if wrong the food will be presented as burnt, overcooked, etc. They will get positive scores as a reinforcement, motivator factor and their positive score will drop with each wrong answer.

In the end, they will receive stars based on their score and will be rewarded with new questions, some guide points that can help them get the correct answer which they can use when a question is asked, and access to higher levels of difficulties.

Of course, many games are not specifically designed to be used in language learning but as a result of creating a fun experience and motivating players (students) to try and understand the story of the game, they help a lot in promoting language learning. Mostly games in RPG (Role Playing Games) and Adventure genres like Fallout series, Divinity, Call of the Sea, etc. Our game is a simple example of how games can help students promote their language skills and motivate them not to give up easily as many do so.

## Results

### Descriptive findings

In this section, first examine the central indicators (mean) and scatter (standard deviation) of research variables:

The results of Table 1 showed that the resilience variable is 155.70 ± 13.31in the experimental group and 159.78 ± 14.71 in the control group. The mean of this variable in the s post-test’experimental group was 188.86 ± 9.93, and in the control group was 159.27 ± 16.99, showing that the score of academic resilience in the experimental group increased after the intervention.

**Table 1:** Central indicators and dispersion of pre-test and post-test in control and experimental groups.

			Experiment group				Control group			
		N	Mean	Std. deviation	Minimum	Maximum	Mean	Std. deviation	Minimum	Maximum
**Pretest**	Academic resilience	37	155.70	13.31	131	180	159.78	14.71	132	187
	Academic self-regulation	37	39.76	3.68	33	47	37.89	5.09	28	50
	Academic achievement	37	136.97	10.18	119	163	141.05	11.89	109	163
**Post-test**	Academic resilience	37	188.86	9.93	163	209	159.27	16.99	120	187
	Academic self-regulation	37	43.68	4.99	34	56	38.16	3.58	31	45
	Academic achievement	37	160.73	6.45	146	174	147.54	11.99	124	169

The mean of the pretest in the academic self-regulation variable is 39.76 ± 3.68 in the experimental group and 37.89 ± 5.09 in the control group. The mean of this variable in the experimental group’s post-test is 43.68 ± 4.99 and in the control group is 38.16 ± 3.58. Academic self-regulation scores increased in the experimental group after the intervention.

The mean of the pre-test in the academic achievement variable is 136.97 ± 10.18 in the experimental group and 141.05 ± 11.89 in the control group, and the mean of this variable in the post-test of the experimental group is 160.73 ± 6.45 and in the control group is 147.54 ± 11.99 which shows the score academic achievement increased in the experimental group after the intervention.

### Inferential analysis

Analysis of covariance was used to test the research hypotheses.

Covariance analysis is a comprehensive type of analysis of variance in which, while comparing the means of one or more groups and estimating one or more independent variables, the effect of one or more intervening variables, or covariate, is excluded from the equation process:

Default 1 – Pre-test: This default is observed, and before implementing the independent variable, covariate (pre-test) has been done.

Defaults 2 and 3 – Normality and homogeneity of variables: The default normality of data was evaluated by the Kolmogorov–Smirnov test and homogeneity test of variance with Leven’s test. The results are as follows:

According to the results of the Kolmogorov–Smirnov test in Table 2, the hypothesis of normality of research variables by control and experimental groups was confirmed (Sig > 0.05). Also, according to Table 2, the Leven test accepted the hypothesis of homogeneity of variances (Sig > 0.05).

**Table 2:** Kolmogorov–Smirnov test (K-S) for pre-test and post-test of control and experimental groups.

		**One-sample Kolmogorov–Smirnov test**				**Test of homogeneity of variances**			
		Experiment group		Control group					
Variable	Period	Test Statistic	Asymp. Sig. (2-tailed)	Test Statistic	Asymp. Sig. (2-tailed)	Levene Statistic	df1	df2	*P* value
Academic resilience	Pre-test	0.070	0.200^*^	0.128	0.128	1.664	1	72	*0.2010*
	Post-test	0.111	0.200^*^	0.095	0.200^*^	3.987	1	72	*0.0520*
Academic self-regulation	Pretest	0.091	0.200^*^	0.137	0.076	3.567	1	72	*0.0630*
	Post-test	0.118	0.200^*^	0.113	0.200^*^	3.178	1	72	*0.0790*
Academic achievement	Pretest	0.107	0.200^*^	0.062	0.200^*^	1.251	1	72	*0.2670*
	Post-test	0.088	0.200^*^	0.097	0.200^*^	1.158	1	72	*0.1680*

*significant level *p* < 0.05.

Default 4: Examining the correlation between research variables.

Pearson test was used to examine the correlation between research-dependent variables. The results are as follows:

The correlation coefficient between the dependent variables of the research was examined in the pre-test and post-test (Table 3). The results showed:

**Table 3:** Correlation coefficients of research variables.

	**Pre-test**			**Post-test**		
	**1**	**2**	**3**	**1**	**2**	**3**
**1-Academic resilience**	–			–		
**2-Academic self-regulation**	0.26^**^	–		0.53^**^	–	
**3-Academic achievement**	0.28^**^	0.53^**^	–	0.63^**^	0.61^**^	–

** Correlation is significant at the 0.01 level (2-tailed).

In the pretest, the correlation coefficient of Academic resilience with Academic self-regulation is equal to 0.26, and with Academic achievement is equal to 0.28 and also the correlation coefficient of Academic achievement and Academic self-regulation is equal to 0.53, all coefficients are significant at the level of 0.01. In the post-test, the correlation coefficient of Academic resilience with Academic self-regulation is 0.53 and with Academic achievement is 0.63, and also the correlation coefficient of Academic achievement and Academic self-regulation is 0.61, all coefficients are significant at the level of 0.01.

To test the results of this hypothesis, a multivariate analysis of covariance is used. The results of the Lambda-Wilkes test are presented in the following table:

The results of Wilkins’s lambda test in Table 4 show that there is a significant difference between the two groups in at least one of the variables (Academic resilience, Academic self-regulation, Academic achievement; *F* = 67,000 and *p* < 0.01). Analyses related to the effects between the subjects were examined, which are shown in Table 4.

**Table 4:** The value of Wilkes’s Lambda test in the analysis of multiple variances of research variables.

**Multivariate tests** ^**a**^						
Effect		Value	F	Hypothesis df	Error df	*P* value
Group	Pillai’s Trace	0.626	37.461^b^	3.000	67.000	*0.001*
	Wilks’ Lambda	0.374	37.461^b^	3.000	67.000	*0.001*
	Hotelling’s Trace	1.677	37.461^b^	3.000	67.000	*0.001*
	Roy’s Largest Root	1.677	37.461^b^	3.000	67.000	*0.001*

a = Design: Intercept, b = Exact statistic.

The following hypotheses are examined:

*Hypothesis 1*: Computer educational games have a significant effect on the academic resilience of third grade (twelfth) female students in Zanjan.

As shown in Table 5, computer educational games have a significant effect on academic resilience (sig = 0.001, *F* = 80.32).

**Table 5:** Comparison of the values of research variables.

**Tests of between-subjects effects**							
Source	Dependent variable	Type III sum of squares	df	Mean square	F	*P* value	Partial eta squared
Group	**Academic resilience**	15358.85	1	15358.85	80.32	*0.000*	0.538
	**Academic self-regulation**	589.07	1	589.07	40.52	*0.000*	0.370
	**Academic achievement**	3553.82	1	3553.82	47.19	*0.000*	0.406
Error	**Academic resilience**	13193.88	69	191.216			
	**Academic self-regulation**	1003.01	69	14.536			
	**Academic achievement**	5196.05	69	75.305			
Total	**Academic resilience**	2272185.74	74				
	**Academic self-regulation**	125824.00	74				
	**Academic achievement**	1767915.11	74				
Corrected total	**Academic resilience**	30138.71	73				
	**Academic self-regulation**	1921.51	73				
	**Academic achievement**	9889.11	73				

Therefore, it was concluded that the mean of the two groups in the post-test after adjusting the pre-test scores was significantly different from each other.

As can be seen in the tables, the mean score of academic resilience in the control group in the pre-test was 159.78 and in the post-test was 159.27, while the mean of this variable in the experimental group (computer training games) in the pre-test was 155.70 and in Post-test was reported to be 188.86.

Due to the significant difference between the scores in the post-test in the control and experimental groups, it was concluded that by eliminating the pre-test factor (Covariate) of computer educational games, reduces academic resilience and according to the size of the effect of the second power factor ETA 54% of the variability of academic resilience in the experimental group is due to computer educational games.

*Hypothesis 2*: Computer educational games have a significant effect on the academic self-regulation of third grade (twelfth) female students in Zanjan.

As shown in Table 5, computer educational games have a significant effect on academic self-regulation (Sig = 0.001, *F* = 40.52).

Therefore, it was concluded that the mean of the two groups in the post-test after adjusting the pre-test scores was significantly different from each other.

As can be seen in the tables, the mean score of academic self-regulation in the control group in the pre-test was 37.89 and in the post-test was 38.16, while the mean of this variable in the experimental group (computer training games) in the pre-test was 39.76 and in the post-test was reported equal to 43.68.

Due to the significant difference between the scores in the post-test in the control and experimental groups, it was concluded that by eliminating the pre-test factor (Covariate) of computer educational games, reduces academic self-regulation.

And due to the size of the effect of the second power factor of ETA,1 to 37% of the educational self-regulatory variability in the experimental group is due to computer educational games.

*Hypothesis 3*: Computer educational games have a significant effect on the academic achievement of third grade (twelfth) female students in Zanjan.

As shown in Table 5, computer educational games significantly affect academic achievement (Sig = 0.001, *F* = 47.19).

Therefore, it was concluded that the mean of the two groups in the post-test after adjusting the pre-test scores was significantly different from each other.

As can be seen in the tables, the mean score of academic achievement in the control group in the pre-test was 141.05 and in the post-test was 147.54, while the average of this variable in the experimental group (computer training games) in the pre-test was 136.97 and in the post-test was reported to be 160/73.

Due to the significant difference between the scores in the post-test in the control and experimental groups, it was concluded that by removing the pre-test factor (Covariate) of computer educational games, reduces academic achievement and according to the size of the effect of the coefficient of quadratic power1 to 40% of the variability of academic achievement in the experimental group is due to computer educational games.

## Discussion

This research aims to evaluate the effect of computer training sessions on academic resilience-academic self-regulation, and academic achievement of female third-grade high school students in Zanjan, Iran. Regarding the first hypothesis Computer educational games significantly affect the academic resilience of third grade (twelfth) female students in Zanjan. As shown in Table 4, computer educational games significantly affect academic resilience. Therefore, it was concluded that the mean of the two groups in the post-test after adjusting the pre-test scores was significantly different from each other. Due to the significant difference between the scores in the post-test in the control and experimental groups, it was concluded that eliminating the pre-test factor (covariate) of computer educational games, reduces academic resilience. Comparison with previous results about the variable of academic resilience is consistent with the results of research (Meneghel et al., 2019; Lepper and Malone, 2021; Singh, 2021; Hunsu et al., 2022; Ononye et al., 2022), and the study was not found to be inconsistent about the variable of academic resilience of this study.

Academic resilience is one of the cognitive (individual) variables affecting academic achievement, which has been one of the academic empowerments and has the greatest impact on academic success (Rudd et al., 2021). The opposite of academic resilience is the lack of conflict or indifference, which implies a lack of effort. Specifically, the teacher should act in the classroom in such a way as to increase students’ involvement in academic success and education(Erbas et al., 2021; Ye et al., 2021). The study of this variable was conducted with the aim that new technologies, particularly educational computers, will increase student engagement and help the teacher in this regard. As observed, the findings in this study indicated that educational computer games could increase academic resilience in students.

Regarding the second hypothesis [Computer educational games have a significant effect on the academic self-regulation of third grade (twelfth) female students in Zanjan]:

The results showed that the level of significance is less than 0.01. Thus, the statistical null hypothesis is rejected. It is found that there is a significant difference between the experimental and control groups in scores related to academic self-regulation in the post-test. It is said that the use of educational computer games has effectively increased the variable of students’ academic self-regulation. Comparison with previous results about the variable of academic resilience is consistent with the results of this research (Zimmerman and Schunk, 2001; Alt and Naamati-Schneider, 2021; Hirt et al., 2021; Jantakun and Jantakoon, 2021; Luo, 2021; Staller et al., 2021; Bransen et al., 2022; Raković et al., 2022). This study was found to be inconsistent in the variable of academic self-regulation (The reason for this inconsistency can be because, firstly, the game used in Soltanizadeh and Bazizadeh’s research is an intellectual and action style game, and secondly, the statistical population of this research is the city of Isfahan, Iran).

According to experts, academic self-regulation is one of the concepts in education in the contemporary world. Today, it is mentioned as an important center and one of the primary axes of education. It is now increasingly used in learning a variety of skills, including cognitive-motor and social skills. It can be said that schools play an essential role in creating their people, and perhaps new technologies can help this responsibility of schools. With this view, the effectiveness of educational computer games was investigated using the game produced on academic self-regulation. The findings were that this technology could affect academic self-regulation. Most previous research also noted this point.

Regarding the third research question [Computer educational games significantly affect the academic achievement of third grade (twelfth) female students in Zanjan].

The result showed that the level of significance for the variable of academic achievement is less than (*p* < 0.01). Thus, the statistical hypothesis of zero is rejected, and it is determined that there is a significant difference between the experimental and control groups in scores related to academic achievement. Based on this, it can be said that educational computer games have effectively increased students’ academic achievement. Comparison with the results of previous about research the variable of academic achievement is consistent with the results of the following study (Zeynali et al., 2019; Abuhassna et al., 2020; Balim et al., 2020; Gökalp, 2020; Ayra and Kösterelioglu, 2021; Şentürk, 2021). Third, the statistical population is different.

Researchers and educational psychologists have always considered academic achievement and the factors affecting it as one of the critical variables in education (Ghory and Ghafory, 2021; Luo, 2021). Perhaps the primary purpose of introducing various technologies and education is to improve students’ progress. According to this point, it was expected that educational computer games would affect academic achievement, which this study confirmed and found that educational computer games also affect academic achievement. According to the present study’s findings and the results of research, it was concluded that computer games can have great potential in education and should not be neglected.

## Limitations

The sample size was small, and samples were limited to female students. Future studies with large sample sizes and both genders will be required to draw better conclusions. Familiarity of the teacher with the application of new technologies in the classroom as well as the cost and time-consuming design of educational computer games can be the limitations of this study.

## Conclusion

This study aimed to investigate the effect of educational computer games on academic resilience, academic self-regulation, and academic achievement of EFL Iranian high school female students in Zanjan, Iran. The results were obtained as follows: Based on the results achieved from the research questions, it can be said that the use of training computer has been effective in increasing the academic resilience of students, increasing their academic self-regulation of the students, and academic achievement of the students.

## Implication

It is suggested that to equip all high schools with computer equipment and hardware – to use a computer or educational games in other courses – to use educational games in different educational levels and combine them with curricula – to improve the quality of teaching – to use computer games in an attractive way of learning – to use computer games to help the teacher to control the classroom – to design and produce supportive games tailored to individual differences - to design and use computer games (educational) to engage students with lesson concepts and to design and use from computer games (educational) in order to turn students into self-regulated individuals.

### Research suggestions

Conducting research in this field on males.

Performing similar research with larger samples.

Carrying out research in this field for a longer period of time.

Carrying out research in this field with other students of different educational levels.

Carrying out research in this field in relation to leading courses of different educational levels.

Carrying out research on the feasibility of using computer games for different groups (teacher, student, parents, etc.)

Carrying out research in relation to various other variables.

## Data availability statement

The raw data supporting the conclusions of this article will be made available by the authors, without undue reservation.

## Author contributions

All authors listed have made a substantial, direct, and intellectual contribution to the work and approved it for publication.

## Funding

This paper was supported by the Youth project of Humanities and social sciences research and planning fund of the Ministry of Education: On the compilation of New-type English-Chinese Medical learner’s Dictionary from the Perspective of Multi-dimensional Definition Theory (18YJC740032).

## Conflict of interest

The authors declare that the research was conducted in the absence of any commercial or financial relationships that could be construed as a potential conflict of interest.

## Publisher’s note

All claims expressed in this article are solely those of the authors and do not necessarily represent those of their affiliated organizations, or those of the publisher, the editors and the reviewers. Any product that may be evaluated in this article, or claim that may be made by its manufacturer, is not guaranteed or endorsed by the publisher.
